# Evaluation of grain yield based on digital images of rice canopy

**DOI:** 10.1186/s13007-019-0416-x

**Published:** 2019-03-22

**Authors:** Kailou Liu, Yazhen Li, Tianfu Han, Xichu Yu, Huicai Ye, Huiwen Hu, Zhihua Hu

**Affiliations:** 1 Jiangxi Institute of Red Soil, National Engineering and Technology Research, Center for Red Soil Improvement; Scientific Observational and Experimental Station of Arable Land Conservation in Jiangxi, Ministry of Agriculture, Nanchang, 331717 People’s Republic of China; 2 Institute of Agricultural Resources and Regional Planning, Chinese Academy of Agricultural Sciences/National Engineering Laboratory for Improving Quality of Arable Land, Beijing, 100081 People’s Republic of China

**Keywords:** Digital camera, Rice canopy, Yield prediction, Late filling stage

## Abstract

**Background:**

Rice canopy changes are associated with changes in the red light (R), green light (G), and blue light (B) value parameters of digital images. To rapidly diagnose the responses of rice to nitrogen (N) fertilizer application and planting density, a simple model based on digital images was developed for predicting and evaluating rice yield.

**Results:**

N application rate and planting density had significant effects on rice yield. Rice yield first increased and then decreased with increasing of N rates, while the rice yield always increased significantly with increasing planting density. The normalized redness intensity (NRI), normalized greenness intensity (NGI), and normalized blueness intensity (NBI) values of the rice canopy varied among stages; however, they were primarily affected by N fertilizer rates, while planting density had no significant effects. Furthermore, the significant relationships of grain yield with NRI and NBI at the late filling stage could be fitted by quadratic equations, but there was no significant relationship observed between grain yield and NGI across all stages. In addition, a field validation experiment showed that the predicted yield based on the fitted quadratic equations was consistent with the measured yield.

**Conclusion:**

The NRI, NGI, and NBI values of rice canopy were mainly affected by N fertilizer rates, while the planting density had no significant effect. The significant relationships between grain yield with NRI and NBI at the late filling stage could be fitted by quadratic equations. Therefore, the canopy NRI and NBI at the late filling stage as measured by digital photography could be used to predict grain yield in southern China.

**Electronic supplementary material:**

The online version of this article (10.1186/s13007-019-0416-x) contains supplementary material, which is available to authorized users.

## Background

As one of the most important food crops in the world, rice feeds half of the global population, especially in East Asia and Southeast Asia [1, 2]. Most rice-dependent populations are currently in developing countries such as China, India, and Thailand [3, 4]. With increasing food demand in these and other regions, advanced technological innovations in rice production will be critical to stabilizing food security.

Rice yield is typically quantified after harvesting and drying. This process requires time, power, and resources. Therefore, the rapid and accurate estimation of rice yield has become an important part of rice production technology research. With the development of remote sensing technology, more remote sensing tools have been applied to agricultural monitoring. Use of terrestrial hyperspectral remote sensing [5, 6] and satellite and aerial imagery [7–9] to conduct rice yield assessment have been reported. At present, the main methods for assessing rice yield primarily include spectroscopic diagnosis [9] and remote sensing evaluation [10], but these methods require the purchase of expensive spectrometers or satellite remote sensing images. Additionally, these methods are complex and give uncertain results [11], which makes them difficult to promote and apply.

However, owing to recent price decreases and integration with smartphones, digital cameras are now widely used [12]. Since the 1990s, digital cameras have been used as one of the most convenient tools for remote sensing of the visible light spectrum (in which R, G, and B represent the gray values from the red, green, and blue channels) in agricultural information monitoring, such as automatic classification of agricultural products [13], weed identification, and pest monitoring [14], among other applications. Kawashima et al. [15] reported manual selection of a complete blade for crop extraction (RB)/(R + B) to estimate the leaf chlorophyll content. Adamsen et al. [16] used a digital camera to vertically capture images of wheat canopies, and cut out images representing 1 m^2^ to calculate parameters such as G/R. That study showed that G/R and the normalized difference vegetation index (NDVI) were significantly related to the chlorophyll metric, which was shown by soil–plant analyses development; (SPAD) value. Rorie et al. [17] used a digital camera to capture images of corn leaves under an active light source and calculated dark green color index (DGCI) after correction with a reference color, with results showing a strong correlation between nitrogen (N) content and yield of leaves. Li et al. [18] vertically photographed wheat canopies and segmented the images using soil adjustment vegetation index (SAVI) Green > 0 and found that the extracted coverage (Canopy Cover) positively correlated with the leaf area index (LAI), plant N content, and aboveground biomass. Previous studies in rice showed that leaf color measured with digital cameras can characterize N and chlorophyll content in rice [19, 20]. In addition, green-channel minus red (GMR) can further distinguish the N diagnosis of i*ndica* and *japonica* rice varieties [21]. In summary, the R, G, and B values of digital photos are closely related to nutrient content in rice [18, 20].

The current applications for digital cameras in rice and other crops are limited to nutritional diagnosis, and only a few studies have reported the evaluation of rice yield with digital cameras [22]. Using digital cameras for predicting rice yields can quickly assess rice production and provide decision-making basis for rice farmers and for governments. In this study, a field experiment was conducted which included different N fertilizer application rates and planting densities, and the color images of the rice canopy in the key growth periods were measured, including values for R, G, and B and the normalized redness intensity (NRI), normalized greenness intensity (NGI), and normalized blueness intensity (NBI). Moreover, the fitted regressions between grain yield with NRI, NGI, and NBI values were established. Field verification was also conducted to evaluate the practicality of the fitted regression.

## Results

### Rice yield

**Fig. 1:**
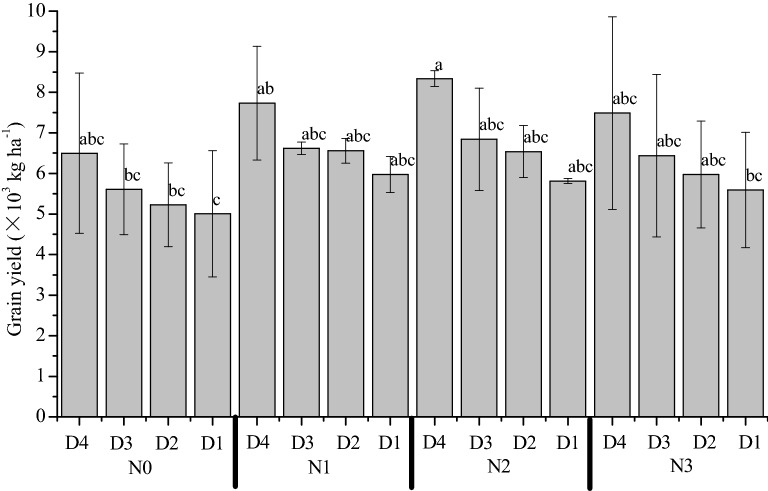
Rice yields of different treatments with varied N and D. Error bar indicates SD. Different lowercase letters indicate significant differences (*p *< 0.05). N represents nitrogen, and D represents planting density

N rates and planting density both had significant effects on rice yield (Fig. 1). Among all treatments, grain yield was highest in the N2D4 treatment with 8.34 × 10^3^ kg ha^−1^, while yield was the lowest in N0D1 treatment (5.01 × 10^3^ kg ha^−1^). Compared with the no N application (N0), the grain yields were higher in the N fertilizer application treatments, but the yields were not increased when N exceeded 180 kg ha^−1^ (N2). There was no significant relationship between N rate and grain yield under the same planting density, as demonstrated through the fitted regressions (Table 1). The fitted regressions showed that grain yield could be significantly improved by increasing planting densities under the same N rate (Table 2). Furthermore, these equations also showed that the growth rate of rice yield was higher in the N2 and N3 treatments than in the N0 and N1 treatments. The yield of the N2 treatment increased by 23.22% over that of the N0 treatment, but the yield of the N1 and N3 treatments did not differ significantly than that of the N0 treatment (Table 3). Increasing rice planting density can significantly improve grain yield. The highest yield was observed in the D4 treatment, which was increased by 34.27% over that of the D1 treatment. However, the yields of D2 and D3 treatments were not significantly increased over that of the D1 treatment.

**Table 1:** The fitted regression between nitrogen (N) rates (x) and grain yield (y)

Planting densities	Fitted equations	*R* ^2^	*p*
D4	y = − 0.0669x^2^ + 20.243x + 6473.3	0.8636	0.42793
D3	y = − 0.0475x^2^ + 14.651x + 5601.5	0.9629	0.16541
D2	y = − 0.0757x^2^ + 20.504x + 5222.3	0.9917	0.11260
D1	y = − 0.0491x^2^ + 13.566x + 5008.5	0.9939	0.17084

D represents planting density

**Table 2:** The fitted regression between planting densities (x) and grain yield (y)

N rates	Fitted equations	*R* ^2^	*p*
N0	y = 8107x + 3153.6	0.9101	0.06309
N1	y = 8881x + 4056.9	0.8804	0.17204
N2	y = 13119x + 2946.8	0.9178	9.56E−04
N3	y = 10260x + 3294.9	0.9387	0.03314

N represents nitrogen

**Table 3:** Comparative analysis of rice yield under nitrogen (N) and density treatments

Treatments	Yield (× 10^3^ kg ha^−1^)	Treatments	Yield (× 10^3^ kg ha^−1^)
N0	5.59 ± 0.67 b	D4	7.51 ± 0.76 A
N1	6.72 ± 0.73 ab	D3	6.38 ± 0.54 B
N2	6.88 ± 1.06 a	D2	6.08 ± 0.63 B
N3	6.37 ± 0.82 ab	D1	5.60 ± 0.42 B

Different lowercase letters indicate significant differences among nitrogen (N) fertilizer treatments (*p *< 0.05). Different uppercase letters indicate significant differences among different planting density treatments (*p *< 0.05)

### Changes in the NRI, NGI, and NBI values of the rice canopy

**Fig. 2:**
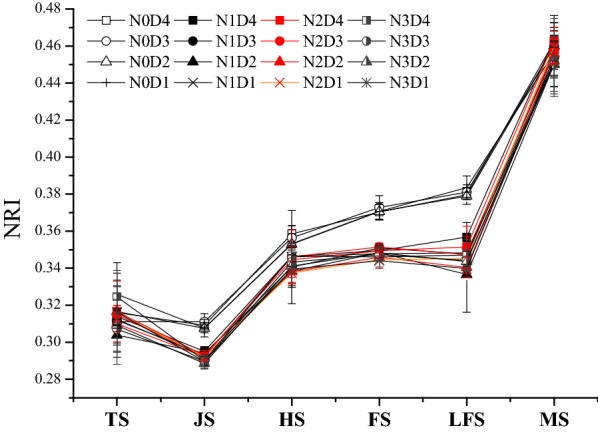
Changes in the normalized redness intensity (NRI) of the rice canopy at key stages among different treatments. Error bar indicates SD. TS, JS, HS, FS, LFS and MS indicate the tillering, jointing, heading, filling, late filling and maturity stages of rice, respectively

The NRI values among all treatments gradually increased with the development of rice growth, which can be roughly divided into three periods (Fig. 2): (1) the gradually increasing period was from the tillering to heading stage, (2) the stable period was from the heading to late filling stage, and (3) the rapidly increasing period was from the late filling to maturity stage. NRI in the key growth stages of rice was primarily regulated by the amount of N fertilizer, and different planting densities have little effect (Table 3). Across different growth periods, the NRI value in the N0 treatment was the highest among the most key stages, although different N rates also caused a significant difference in NRI values. Compared with the no N fertilizer treatment (Table 4), the NRI values in the N1 treatment were reduced by 4.86%, 3.51%, and 5.91% in the heading, filling, and late filling stages, respectively, while those in the N2 treatment decreased by 5.31%, 3.88%, and 6.27%, and those of the N3 treatment decreased by 6.25%, 3.57%, and 6.65% across these same stages.

**Table 4:** Comparative analysis of the normalized redness intensity (NRI) in rice canopy under nitrogen (N) fertilizer and density treatments

Treatments	TS	JS	HS	FS	LFS	MS
N0	0.317 ± 0.006a	0.309 ± 0.002a	0.355 ± 0.003a	0.371 ± 0.001a	0.381 ± 0.002a	0.460 ± 0.002a
N1	0.310 ± 0.004a	0.294 ± 0.001b	0.343 ± 0.004b	0.349 ± 0.001b	0.347 ± 0.008b	0.456 ± 0.006a
N2	0.314 ± 0.003a	0.292 ± 0.000b	0.342 ± 0.004b	0.348 ± 0.003b	0.346 ± 0.005b	0.459 ± 0.003a
N3	0.314 ± 0.008a	0.290 ± 0.001b	0.343 ± 0.003b	0.346 ± 0.002b	0.344 ± 0.003b	0.452 ± 0.002a
D4	0.322 ± 0.005A	0.297 ± 0.008A	0.347 ± 0.006A	0.353 ± 0.011A	0.356 ± 0.014A	0.456 ± 0.006A
D3	0.312 ± 0.003A	0.297 ± 0.010A	0.345 ± 0.007A	0.355 ± 0.012A	0.353 ± 0.017A	0.454 ± 0.004A
D2	0.308 ± 0.005A	0.296 ± 0.008A	0.340 ± 0.007A	0.352 ± 0.012A	0.349 ± 0.020A	0.454 ± 0.004A
D1	0.316 ± 0.006A	0.296 ± 0.008A	0.345 ± 0.007A	0.352 ± 0.013A	0.354 ± 0.020A	0.459 ± 0.003A

TS, JS, HS, FS, LFS and MS indicate the tillering, jointing, heading, filling, late filling and maturity stages of rice, respectively. Different lowercase letters indicate significant differences among nitrogen (N) fertilizer treatments (*p *< 0.05). Different uppercase letters indicate significant differences among planting density treatments (*p *< 0.05)

**Fig. 3:**
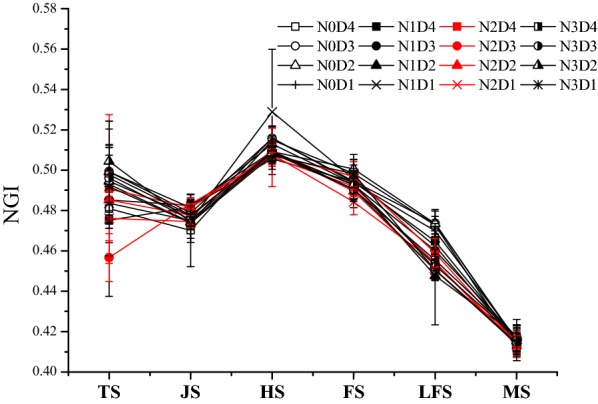
Changes in the normalized greenness intensity (NGI) of the rice canopy at key stages among different treatments. Error bar indicates SD. TS, JS, HS, FS, LFS and MS indicate the tillering, jointing, heading, filling, late filling and maturity stages of rice, respectively

The NGI values of all treatments gradually decreased with increasing rice growth, and these can be divided into three general periods as well (Fig. 3): (1) a stable period was from the tillering to the jointing stage, (2) a gradual increase period was from the jointing to the heading stage, and (3) a rapid reduction period was from the heading to the maturity stage. Similar to trends for NRI, NGI during the key growth stages of rice could be affected by the N rates, but different planting densities had little effect (Table 4). Among four N rates, the NGI value of the tillering stage was the highest in the N3 treatment. However, in the late filling stage, the NGI values for the N fertilizer treatments were significantly lower than that of the N0 treatment (Table 5). The NGI values in the N1, N2, and N3 treatments decreased below that of the N0 treatment by 2.90%, 3.82%, and 4.26%, respectively.

**Table 5:** Comparative analysis of the normalized greenness intensity (NGI) in rice canopy under nitrogen (N) fertilizer and density treatments

Treatments	TS	JS	HS	FS	LFS	MS
N0	0.492 ± 0.008ab	0.475 ± 0.004a	0.514 ± 0.003a	0.497 ± 0.003a	0.472 ± 0.002a	0.415 ± 0.002a
N1	0.486 ± 0.010b	0.480 ± 0.004a	0.514 ± 0.010a	0.494 ± 0.001a	0.459 ± 0.008b	0.416 ± 0.001a
N2	0.477 ± 0.015ab	0.479 ± 0.004a	0.508 ± 0.001a	0.491 ± 0.005a	0.454 ± 0.003b	0.414 ± 0.001a
N3	0.498 ± 0.005a	0.475 ± 0.002a	0.508 ± 0.001a	0.493 ± 0.004a	0.452 ± 0.002b	0.416 ± 0.002a
D4	0.479 ± 0.010A	0.478 ± 0.005A	0.509 ± 0.004A	0.496 ± 0.003A	0.462 ± 0.008A	0.417 ± 0.002A
D3	0.481 ± 0.019A	0.480 ± 0.004A	0.510 ± 0.004A	0.492 ± 0.002A	0.458 ± 0.010A	0.417 ± 0.002A
D2	0.494 ± 0.008A	0.479 ± 0.003A	0.512 ± 0.002A	0.496 ± 0.005A	0.456 ± 0.012A	0.417 ± 0.001A
D1	0.484 ± 0.008A	0.474 ± 0.000A	0.515 ± 0.010A	0.492 ± 0.006A	0.458 ± 0.009A	0.415 ± 0.001A

TS, JS, HS, FS, LFS and MS indicate the tillering, jointing, heading, filling, late filling and maturity stages of rice, respectively. Different lowercase letters indicate significant differences among nitrogen (N) fertilizer treatments (*p *< 0.05); different uppercase letters indicate significant differences among planting density treatments (*p *< 0.05)

**Fig. 4:**
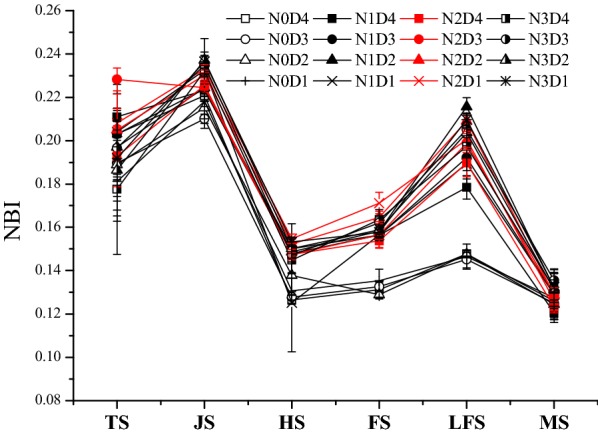
Changes in the normalized blueness intensity (NBI) of the rice canopy at key stages among different treatments. Error bar indicates SD. TS, JS, HS, FS, LFS and MS indicate the tillering, jointing, heading, filling, late filling and maturity stages of rice, respectively

Across all treatments, NBI values gradually decreased along with rice growth, and could be roughly divided into four periods, which include: (1) the gradual increase period was from the tillering to the jointing stage, (2) the rapid reduction period was from the jointing to the heading stage, (3) the increase period was from the heading to the late filling stage, and (4) the stabilization period was from the late filling to the maturity stage (Fig. 4). Like NRI and NGI, the NBI values in the key growth stages of rice were also affected by N rates (Table 5), while the different planting densities had no significant effect. There was no significant difference for NBI in the maturity stage across different treatments. In the tillering stage, the NBI values of the N1 and N2 treatments were significantly higher than for the N0 and N3 treatments, but in all other stages, the NBI values in the N0 treatment were the lowest. However, compared with the N0 treatment, the NBI values were significantly higher in the N fertilizer (N1, N2, and N3) treatments (Table 6). In the jointing, heading, filling, and late filling stages, the NBI values of the N1 treatment decreased below that of the N0 treatment by 4.62%, 9.37%, 18.81%, and 32.18%, respectively. The NBI values of the N2 treatments were reduced by 5.68%, 15.12%, 22.30%, and 35.99%, respectively, and the NBI values of the N3 treatments were reduced by 8.88%, 13.90%, 21.59%, and 39.03%, respectively.

**Table 6:** Comparative analysis of the normalized blueness intensity (NBI) in rice canopy under nitrogen (N) fertilizer and density treatments

Treatments	TS	JS	HS	FS	LFS	MS
N0	0.191 ± 0.009 b	0.216 ± 0.005c	0.131 ± 0.005b	0.132 ± 0.003b	0.147 ± 0.001b	0.126 ± 0.001a
N1	0.204 ± 0.006 a	0.226 ± 0.004b	0.143 ± 0.012a	0.157 ± 0.001a	0.194 ± 0.016a	0.128 ± 0.005a
N2	0.209 ± 0.015 a	0.228 ± 0.004b	0.150 ± 0.004a	0.162 ± 0.008a	0.200 ± 0.008a	0.127 ± 0.003a
N3	0.188 ± 0.008b	0.235 ± 0.002a	0.149 ± 0.004a	0.161 ± 0.003a	0.204 ± 0.005a	0.133 ± 0.002a
D4	0.199 ± 0.014A	0.226 ± 0.005A	0.144 ± 0.010A	0.151 ± 0.014A	0.182 ± 0.022A	0.126 ± 0.005A
D3	0.207 ± 0.017A	0.223 ± 0.010A	0.145 ± 0.010A	0.153 ± 0.013A	0.188 ± 0.027A	0.128 ± 0.004A
D2	0.197 ± 0.008A	0.225 ± 0.009A	0.148 ± 0.006A	0.152 ± 0.016A	0.195 ± 0.031A	0.129 ± 0.003A
D1	0.200 ± 0.012A	0.230 ± 0.008A	0.141 ± 0.015A	0.156 ± 0.015A	0.188 ± 0.028A	0.125 ± 0.003A

TS, JS, HS, FS, LFS and MS indicate the tillering, jointing, heading, filling, late filling and maturity stages of rice, respectively. Different lowercase letters indicate significant differences among nitrogen (N) fertilizer treatments (*p *< 0.05); different uppercase letters indicate significant differences among planting density treatments (*p *< 0.05)

### Relationships between rice yield and NRI, NBI, and NGI

Across different key growth stages of rice, the relationships between yield and NRI or NBI of rice canopy digital images at the late filling stage were fitted by quadratic curves (Table 7), with y = − 3437.4x^2^ + 2465.5x − 434.79 (*R*^2^ = 0.4853, *p *< 0.05); and y = − 1198.9x^2^ + 436.5x − 32.612 (*R*^2^ = 0.4122, *p *< 0.05), respectively. However, there was no significant relationship between NGI and yield.

**Table 7:** Fitted equations for the normalized redness intensity (NRI), normalized greenness intensity (NGI), and normalized blueness intensity (NBI) values (x) and yield (y) across different growth stages of rice

Stages	NRI			NGI			NBI		
	Regression equations	*R* ^2^	*p*	Regression equations	*R* ^2^	*p*	Regression equations	*R* ^2^	*p*
TS	y = − 2E + 06x^2^ + 1E + 06x − 188418	0.0109	0.9311	y = − 119334x^2^ + 96588x − 12308	0.0597	0.67032	y = 245939x^2^ − 80957x + 12745	0.0664	0.63984
PS	y = − 9E + 06x^2^ + 5E + 06x − 783277	0.2824	0.11566	y = 2E + 07x^2^ − 2E + 07x + 4E + 06	0.3632	0.05322	y = − 8E + 06x^2^ + 4E + 06x − 403687	0.3531	0.05894
HS	y = − 8E + 06x^2^ + 6E + 06x − 976732	0.1738	0.28902	y = 7E + 06x^2^ − 7E + 06x + 2E + 06	0.226	0.18915	y = − 4E + 06x^2^ + 1E + 06x − 73035	0.1926	0.24896
FS	y = − 9E + 06x^2^ + 6E + 06x − 1E + 06	0.4148	0.03074	y = − 250780x^2^ + 158991x − 10957	0.1883	0.46575	y = − 2E + 06x^2^ + 755848x − 51130	0.293	0.10504
LFS	y = − 3E + 06x^2^ + 2E + 06x − 434790	0.4853	0.01333	y = − 158882x^2^ + 99109x − 5580.9	0.2439	0.10197	y = − 1E + 06x^2^ + 436500x − 32612	0.4122	0.03163

TS, JS, HS, FS, LFS and MS indicate the tillering, jointing, heading, filling, late filling and maturity stages of rice, respectively

### Model verification

**Fig. 5:**
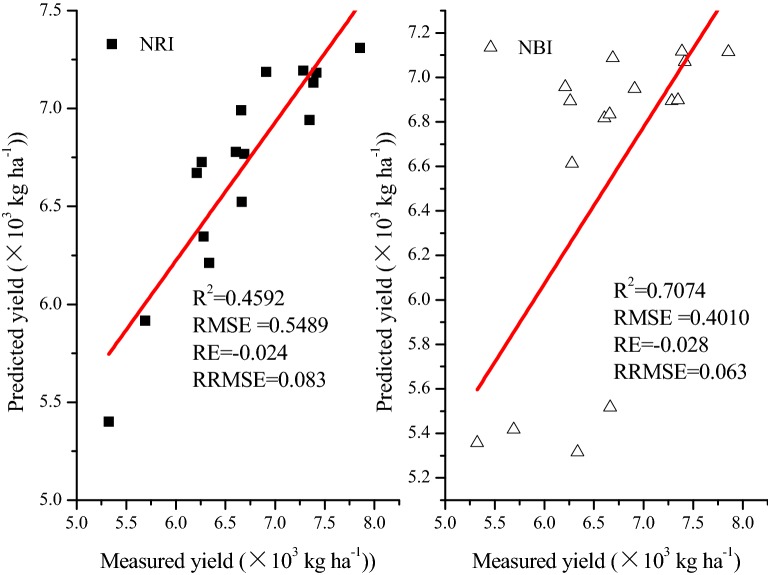
The relationships between predicted and measured yield. R^2^, RMSE, RE and RRMSE mean correlation coefficient, root mean square error, relative error and relative root mean square error, respectively. NRI and NBI indicate the normalized redness intensity and normalized blueness intensity

In field validation experiments, the predicted yields were attained through the fitted equations with NRI and NBI at the late filling stage (Fig. 5). Concurrence between the simulated and measured values was high, with *R*^2^ of 0.4592 and 0.7074 and RMSE of 0.5489 and 0.4010, respectively, with average RE values of − 0.024 and − 0.028. Therefore, the NRI and NBI at the late filling stage can be used to better predict grain yield.

## Discussion

The application of digital cameras and image processing technology can be used to quickly obtain crop canopy data, and this method is cheaper and easier to operate and implement than other methods, such as hyperspectral remote sensing and satellite imagery. The rapid increase in the use of smartphones (with a self-contained camera function) can enable this technology to meet huge potential applications for rice planting. In this study, with increases in rice growth (from the tillering to the maturity stage), NRI values increased gradually, while NGI and NBI peaked at the heading and jointing stages, respectively, and then gradually decreased. This may be due to growth and metabolism consuming a great deal of energy, because NRI is directly related to available energy [23, 24]. Moreover, NRI, NGI, and NBI in key growth stages of rice were mainly regulated by N fertilizer application, rather than by planting density. Compared with no N application, the NRI values of the heading stage in the N fertilizer treatments were significantly lower, but the NBI values were significantly higher. The R, G, B values could change as a result of leaf yellowing and senescence, which can be caused by insufficient nutrient supply [25, 26].

The R, G, and B values of plant canopies can reflect nutrient uptake, especially that of N content and uptake by crops [17–19]. Previous studies have shown that for rice, wheat, corn, and cotton, the R, G, B values obtained by a camera can reflect the N uptake capacity of plants, and can be further used to diagnose N deficiency and assess biomass and grain yield [18, 20, 22]. The current study also found that the relationship between NRI or NBI and grain yield can be fitted by a quadratic curve across all key rice growth stages. This result was the same as other studies, which all suggested that grain yield was closely related to NRI and NBI [27, 28]. In these field experiments, the rainfall and temperature that occurred in the second year (2017) of the verification experiments were similar to those of the first year (2016). The simulated values of rice yield obtained by fitting equations were highly consistent with the measured values, with high estimation accuracy and low average relative error. NRI and NBI at the late filling stage can thus be used to better predict grain yield. However, rice yield could change as a result of the interaction of complex factors [29, 30], such as unusual precipitation and temperature, which can cause rice lodging and disease [31–33]. Therefore, actual rice yield may differ from the yield predicted by NRI and NBI at the late filling stage, so these predictions should be used cautiously.

## Conclusion

Metrics of growth in rice canopy across different growth stages can be measured and assessed by changes to the R, G, and B parameters of digital images. The NRI, NGI, and NBI of the rice canopy varied significantly across different N fertilizer rates, though the measured rice yield varied depending on both N fertilizer rate and planting density. NRI and NBI at the late filling stage could be used to predict grain yield through using the fitted quadratic curve equations, and these results were upheld by the field validation experiment.

## Methods

### Site description

**Fig. 6:**
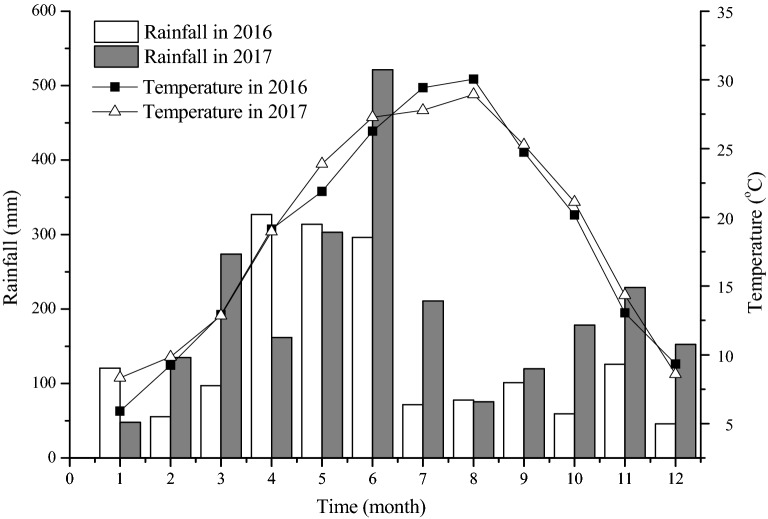
Meteorological data for the field experimental site in 2016 and 2017

The field experiment was conducted at Yanjia Village, Zhanggong Town, Jinxian County, Nanchang City, Jiangxi Province, China (116°′24″E, 28°15′30″N). This area experiences a mid-subtropical monsoon climate, with an average annual rainfall of 1537 mm, annual evaporation of 1150 mm, annual average temperature of 18.1 °C, with average temperatures in the coldest month (January) and the hottest month (July) of 4.6 °C and 29.1 °C, respectively. In the experimental years of the study (2016 and 2017), rainfall primarily occurred in March, April, May, and June (Fig. 6). The proportion of rainfall received in this season was 61.14% for 2016 and 52.34% for 2017. The rice growing season (from July to November) experiences higher temperature and lower rainfall. Average temperatures from July to November were 23.49 °C and 23.49 °C in 2016 and 2017, respectively, and the total rainfalls were 435.40 mm and 812.90 mm. The altitude is 25–30 m, a typical low hilly area. The soil type is paddy soil developed by Quaternary red clay. Soil pH is 6.9, the organic carbon is 16.22 g kg^−1^, total N is 0.95 g kg^−1^, total phosphorus is 1.02 g kg^−1^, total potassium is 15.41 g kg^−1^, and alkaline N is 143.70 mg kg^−1^; available phosphorus is 10.30 mg kg^−1^, and available potassium is 125.10 mg kg^−1^.

### Experimental design

In this study, the field experiment was conducted in 2016 and 2017 for model establishment and validation, respectively. The main treatment was N fertilizer rates (0, 135, 180, and 225 kg ha^−1^ N) and the secondary treatment was density levels (0.21, 0.27, 0.33 and 0.39 million plants ha^−1^). Each treatment was three replications, and main area was 9 × 4.5 = 40.5 m^2^ (the sub-area was 2.2 × 4.5 = 9.9 m^2^), and the random area group was arranged.

Chemical fertilizer was applied to treatments (Table 8). The application ratios for N fertilizer were 40%, 30%, and 30% in basal, tiller, and panicle fertilizers, respectively. All treatments (including the no N fertilizer treatment) received application of 60 kg ha^−1^ P_2_O_5_ with calcium magnesium phosphorus (12.5% P_2_O_5_) and 225 kg ha^−1^ K_2_O with potassium chloride (60% K_2_O). All phosphorus and 50% of the potassium fertilizer were used as base fertilizer, while the remaining 50% potassium fertilizer was applied as panicle fertilizer. The application time for basal, tiller, and panicle fertilizers was 1 day before transplanting rice, 10 days and 45 days after transplanting rice, respectively.

**Table 8:** The nitrogen (N) fertilizer rates and planting density in different treatments

Treatments	N fertilizer rates (kg ha^−1^)	Planting densities (million plants ha^−1^)	Plant spacing (× 10^−2^ m)	Line spacing (× 10^−2^ m)
N0D1	0	0.21	20	23.8
N0D2	0	0.27	20	18.5
N0D3	0	0.33	20	15.2
N0D4	0	0.39	20	12.8
N1D1	135	0.21	20	23.8
N1D2	135	0.27	20	18.5
N1D3	135	0.33	20	15.2
N1D4	135	0.39	20	12.8
N2D1	180	0.21	20	23.8
N2D2	180	0.27	20	18.5
N2D3	180	0.33	20	15.2
N2D4	180	0.39	20	12.8
N3D1	225	0.21	20	23.8
N3D2	225	0.27	20	18.5
N3D3	225	0.33	20	15.2
N3D4	225	0.39	20	12.8

N represents nitrogen; and D represents planting density

The rice variety was ‘Zhengcheng 456’, which was sown on 25th June, transplanted on 24th July, harvested on 5th November. Water, weeds, insects, and disease were controlled as required to avoid yield loss.

### Measurement index

1. Rice yield determination

Matured rice plants in each plot were harvested for threshing and measured for standard yield after drying (water content was 13.5%).

2) Photographing rice canopy

In the tillering, jointing, heading, filling, late filling, and maturity stages, images of the rice canopy of rice in 2016 and 2017 (Additional file 1: Figs. S1 and S2) were obtained in the field with a Canon IXUS140 digital camera following established methods [34]. Crops were photographed at a vertical height of 1.2 m from the ground (about 1 m from the rice canopy) and at a 60° angle to the ground. A 15 × 5 cm white plastic plate was used as the background for shooting in the camera’s automatic white balance mode. The image resolution was 1280 × 960, and the camera’s image of the ground rice canopy range was approximately 1.2 m × 1 m trapezoids. Digital images were transferred to the computer in JPEG format.

The image was processed using Adobe Photoshop. “Color selection” was used to select the plant canopy part of the digital image (without the interference of the soil or water surface), and then the “histogram procedure” was employed to obtain data. The R, G, and B values of the image were measured, and the corresponding NRI, NGI, and NBI were calculated. The calculation of each normalized value is as follows:

$${\text{NRI}} = {\text{R}}/\left( {{\text{R}} + {\text{G}} + {\text{B}}} \right)$$

$${\text{NGI}} = {\text{G}}/\left( {{\text{R}} + {\text{G}} + {\text{B}}} \right)$$

$${\text{NBI}} = {\text{B}}/\left( {{\text{R}} + {\text{G}} + {\text{B}}} \right)$$

3) Statistical analysis and model validation

The yield difference between treatments was statistically analyzed in SPSS16.0. Differences were compared with the Duncan method, with differences in N fertilizer application rate and planting density distinguished. When *p *< 0.05, the difference was significant.

The evaluation model was constructed through linear relationships between grain yield and color parameters through data of 2016. In order to test the reliability and universality of the model, the established models were validated using the data of 2017. The validity of the models was estimated from the statistical values of RMSE (root mean square error), RE (relative error), and RRMSE (relative root mean square error), which were calculated as:

1 $$RMSE = \sqrt {\frac{{\sum {\left( {X_{0} - X_{S} } \right)^{2} } }}{n}}$$

2 $$RE = \frac{{X_{0} - X_{S} }}{{X_{O} }} \times 100\%$$

3 $$RRMSE = \sqrt {\frac{1}{n}\sum {\left( {\frac{{X_{s} - X_{0} }}{{X_{0} }}} \right)^{2} } }$$

where X_0_ and X_S_ represent measured and predicted values, respectively. The model is available when RRMSE < 25%.

## Additional file

**Additional file 1: Fig. S1.** The rice growth of tillering (A), jointing (B), heading (C), filling (D), late filling (E) and maturity (F) stages in 2016. **Fig. S2** The rice growth of late filling stage in 2017.

## Abbreviations

R: red light
G: green light
B: blue light
N: nitrogen
NRI: normalized redness intensity
NGI: normalized greenness intensity
NBI: normalized blueness intensity
NDVI: normalized difference vegetation index
SPAD: soil-plant analyses development
SAVI: soil adjustment vegetation index
DGCI: dark green color index
LAI: leaf area index
GMR: green-channel minus red
RMSE: root mean square error
RE: relative error
RRMSE: relative root mean square error
