# Supplementary material for: Evaluation of grain yield based on digital images of rice canopy
Source: Plant Methods. 2019 Mar 22;15:28. doi: 10.1186/s13007-019-0416-x (PMC6429754; doi:10.1186/s13007-019-0416-x)
Supplement: Supplementary file 1 — Additional file 1: Fig. S1. The rice growth of tillering (A), jointing (B), heading (C), filling (D), late filling (E) and maturity (F) stages in 2016. Fig. S2 The rice growth of late filling stage in 2017. [file 13007_2019_416_MOESM1_ESM.doc]

Additional file


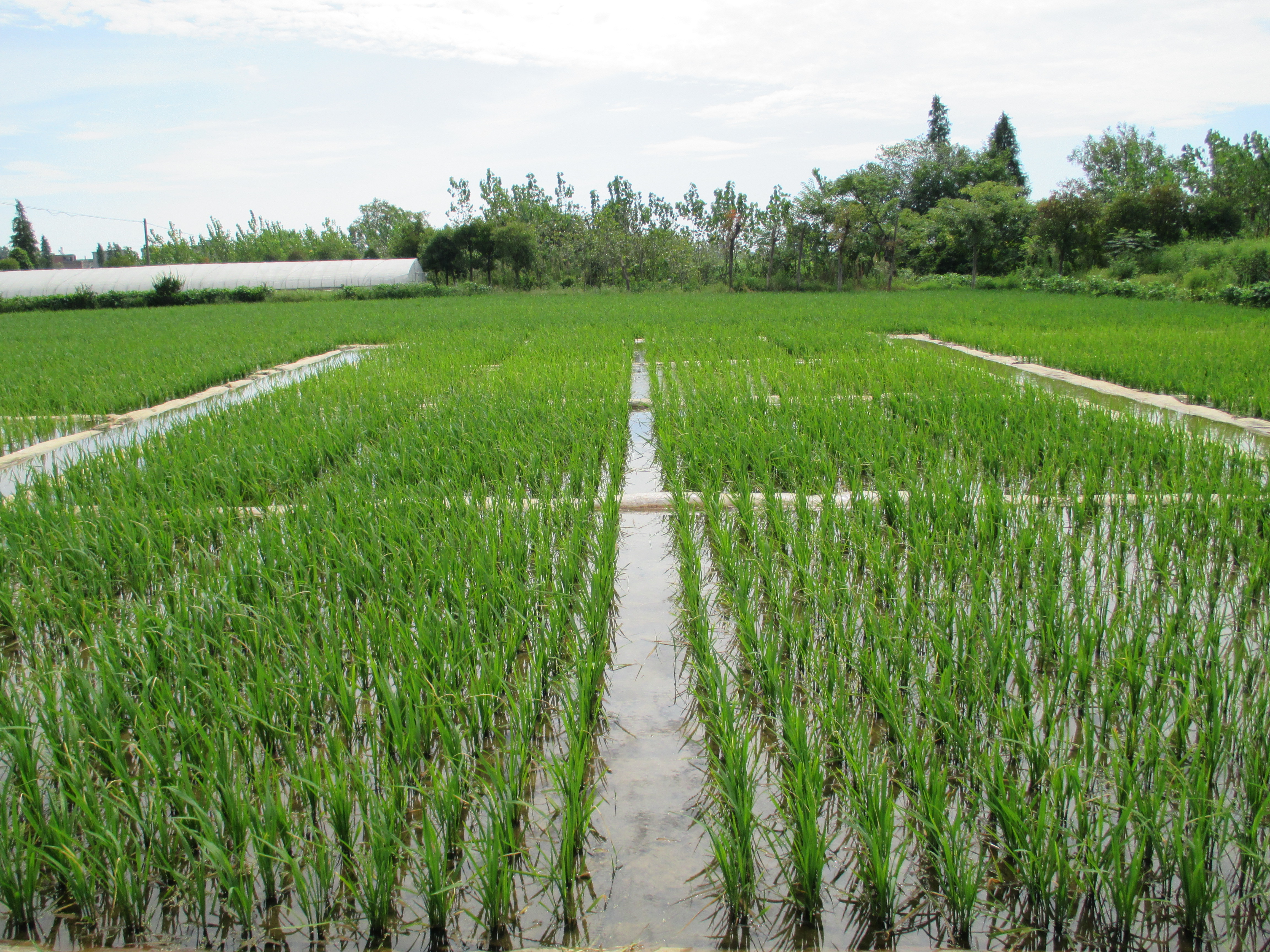

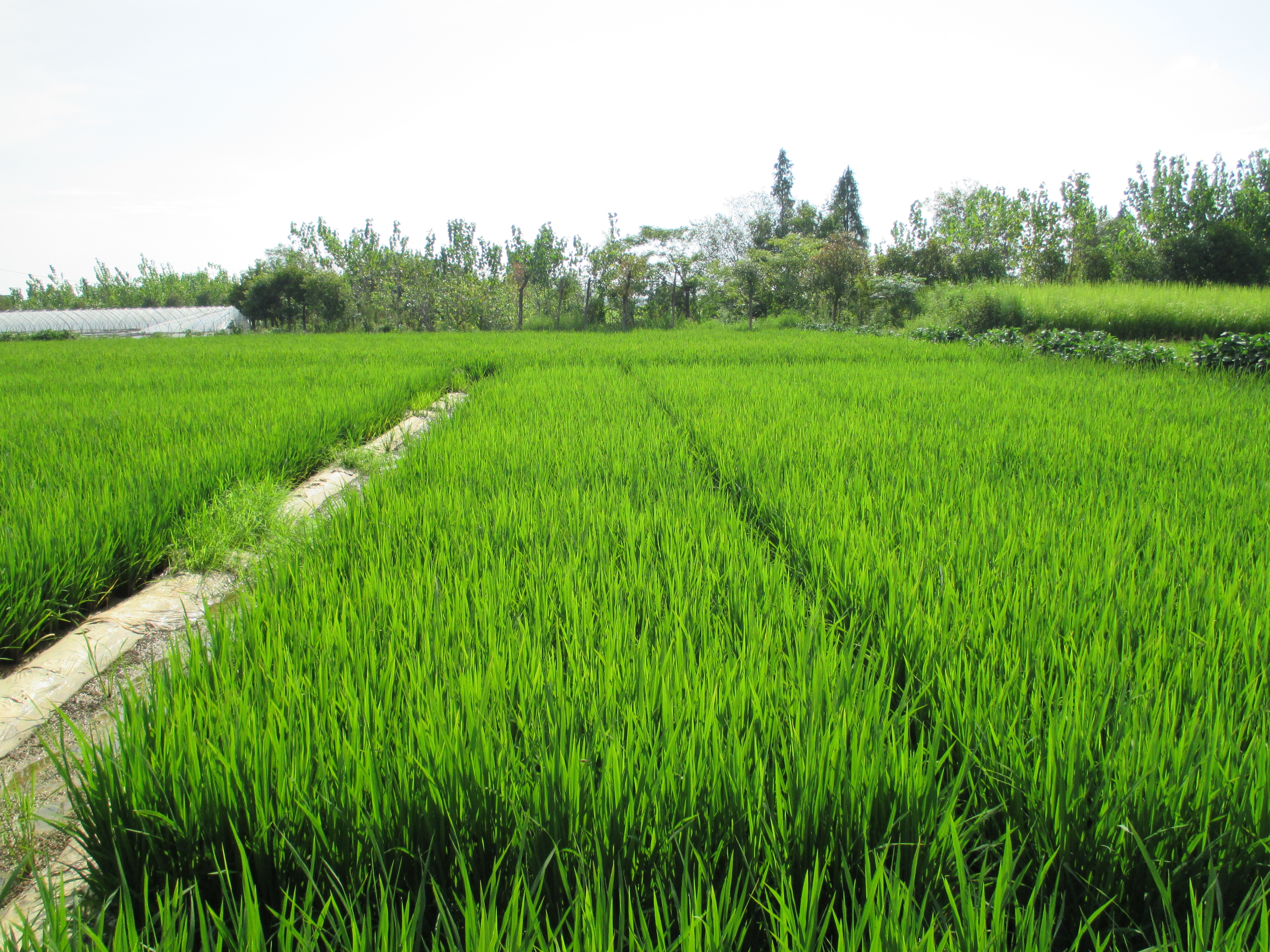


B


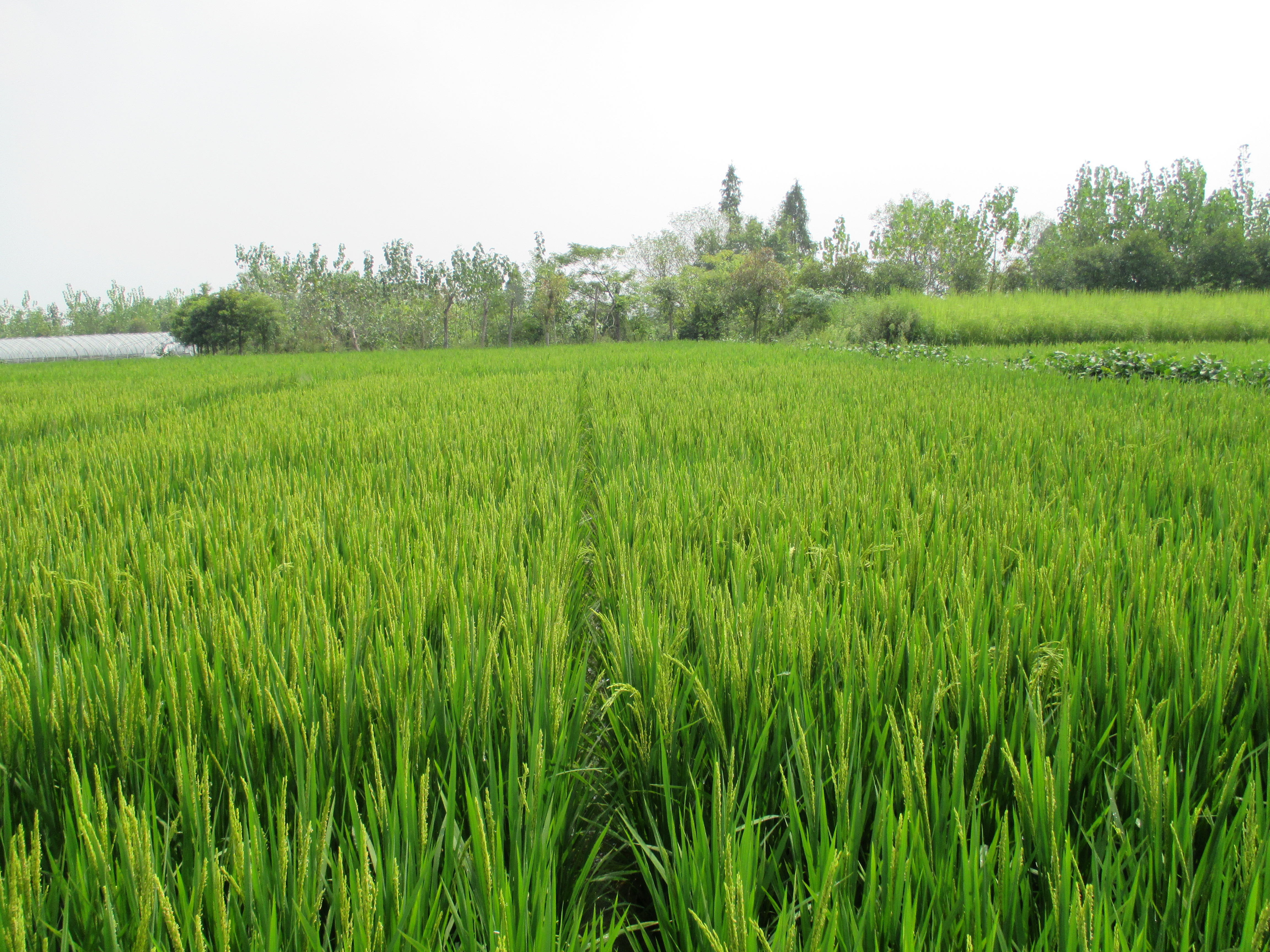

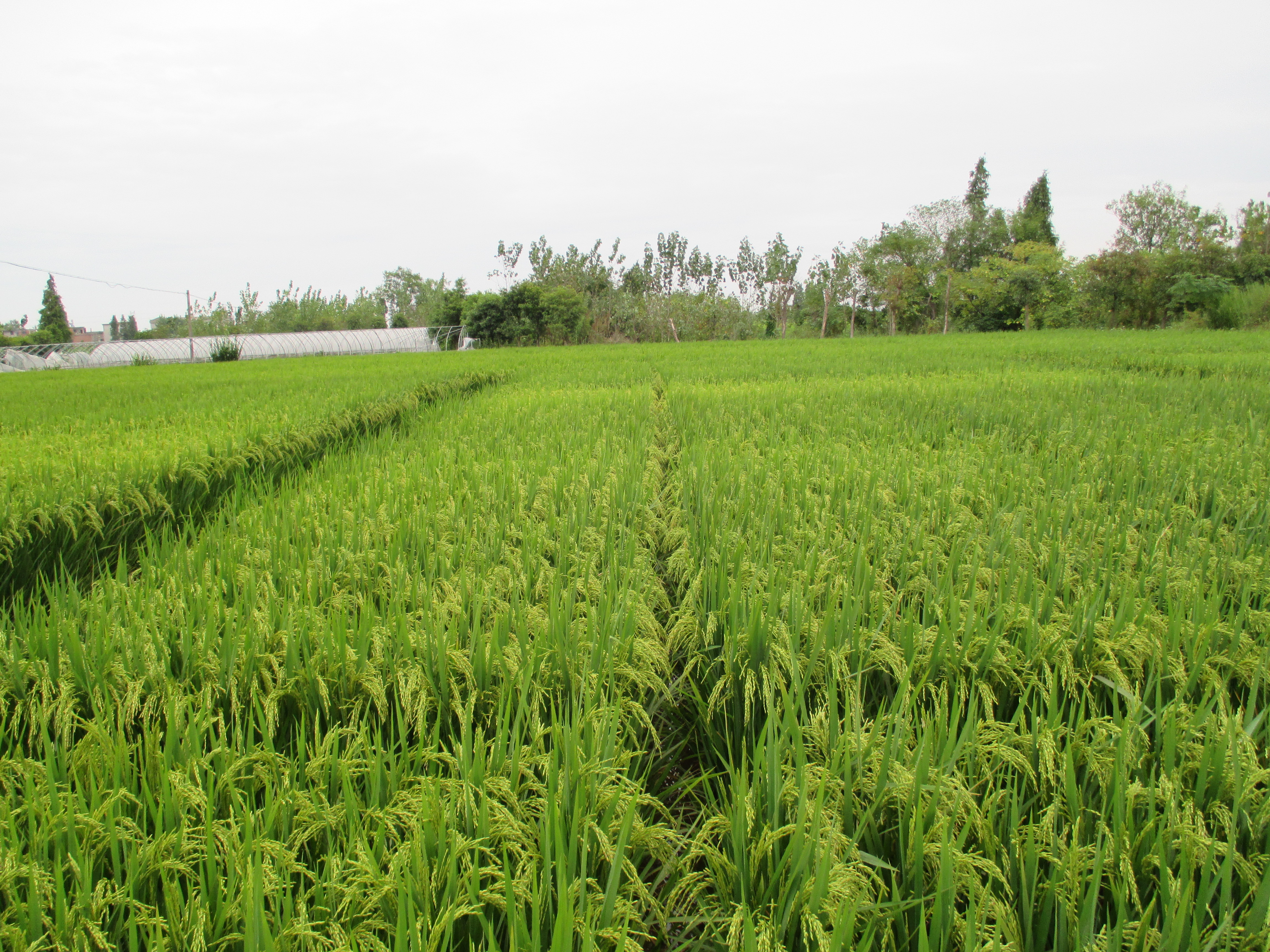


D

C


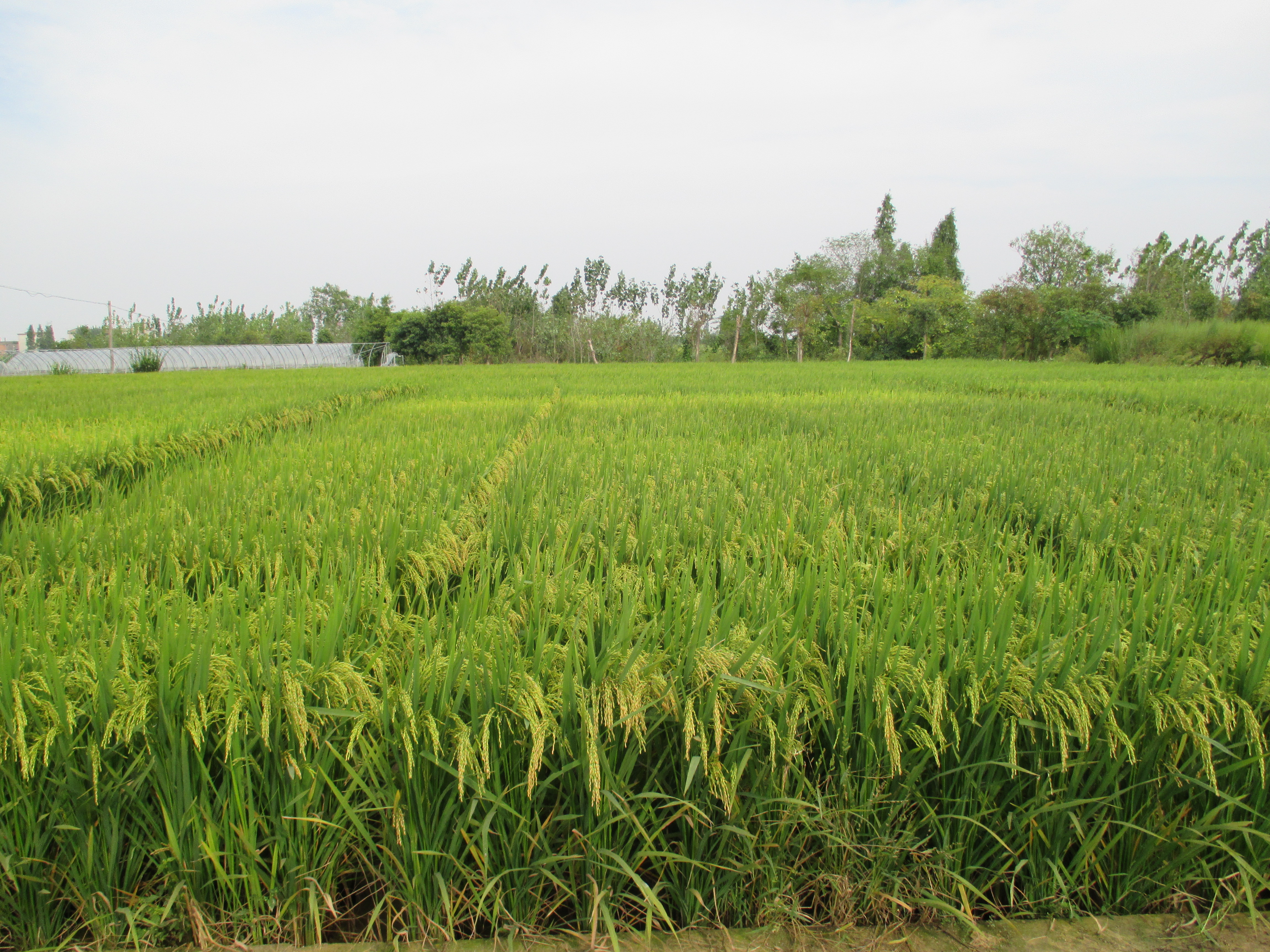

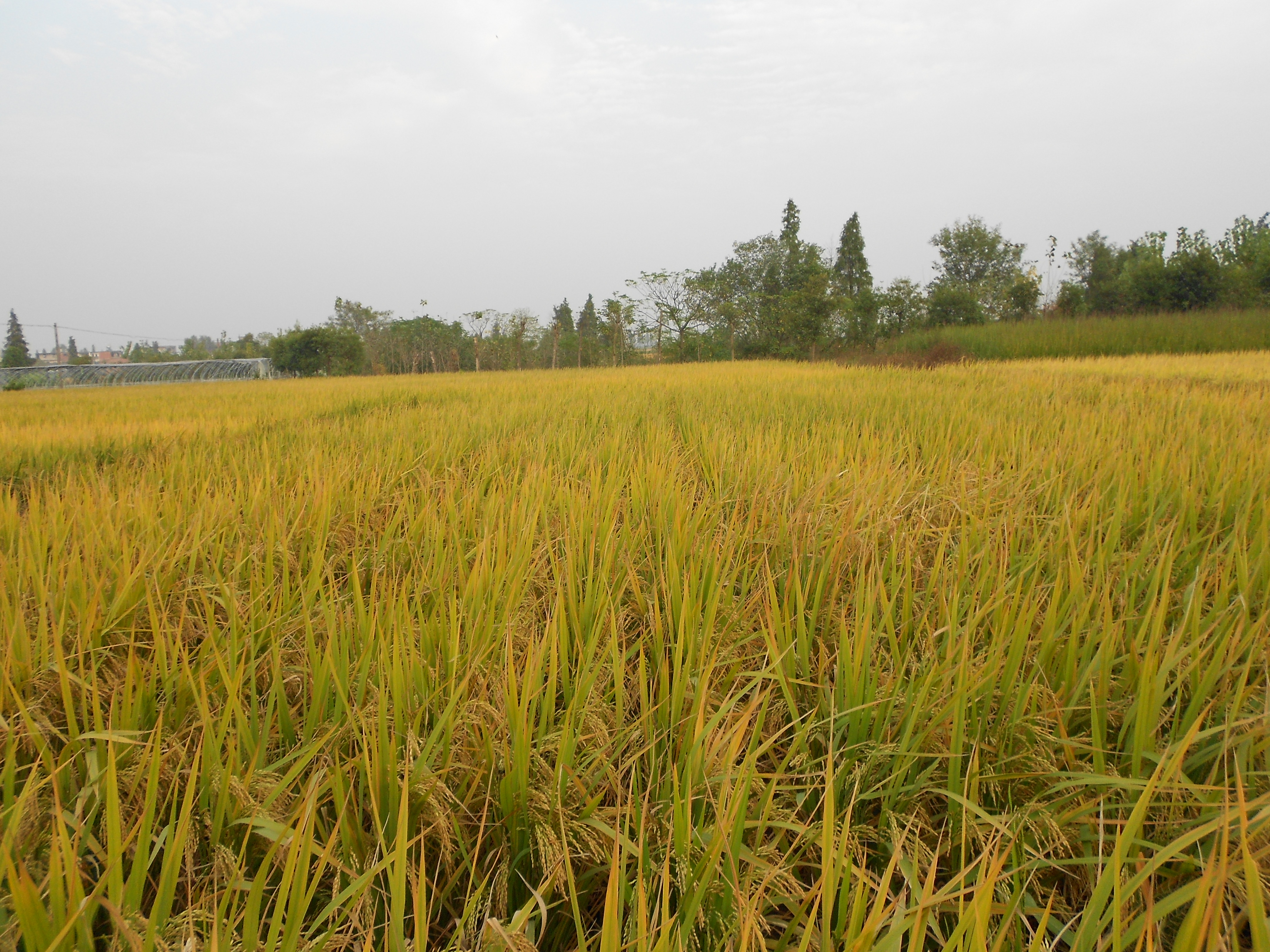


F

E

Fig. S1 The rice growth of tillering (A), jointing (B), heading (C), filling (D), late filling (E) and maturity (F) stages in 2016

A


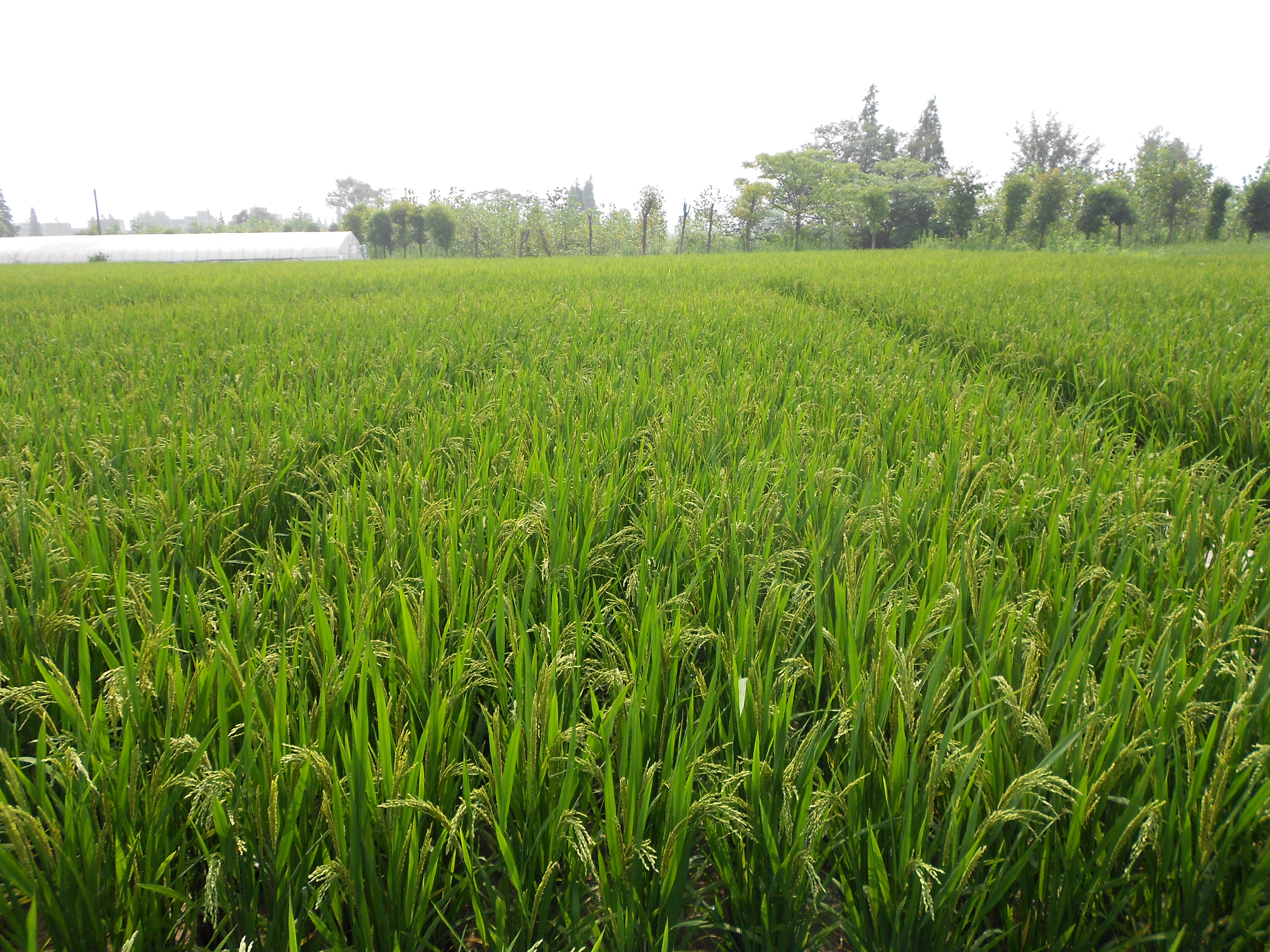


Fig. S2 The rice growth of late filling stage in 2017
